# *Salmonella* Typhimurium Outbreak Associated with Veterinary Clinic

**DOI:** 10.3201/eid1012.040714

**Published:** 2004-12

**Authors:** Bryan Cherry, Amy Burns, Geraldine S. Johnson, Heidi Pfeiffer, Nellie Dumas, Donna Barrett, Patrick L. McDonough, Millicent Eidson

**Affiliations:** * New York State Department of Health, Albany, New York, USA;; † Madison County Department of Health, Wampsville, New York, USA;; ‡ Cornell University College of Veterinary Medicine, Ithaca, New York, USA

**Keywords:** Salmonella Typhimurium, salmonellosis, veterinary clinic, Salmonella infections, dispatch

## Abstract

A *Salmonella enterica* serovar Typhimurium outbreak was associated with a veterinary clinic. Confirmed cases were in one cat, two veterinary technicians, four persons associated with clinic patients, and a nurse not linked to the clinic. This outbreak emphasizes the importance of strong public health ties to the animal health community.

Zoonotic transmission of *Salmonella enterica* has been associated with exposure to sick and healthy cattle on farms (*1*), sick cats at animal shelters (*2*), and cats at small animal veterinary clinics (*2*). Salmonellosis is a well-recognized nosocomial problem at large-animal veterinary hospitals (*3**,**4*), but it is associated with few, if any, human outbreaks. In small-animal medicine, salmonellosis is likely underrecognized because gastrointestinal illness is common and often self-limiting. As in human medicine, salmonellosis is rarely confirmed in a laboratory, which results in underreporting of cases. However, in 1999 two salmonellosis outbreaks at veterinary clinics were linked to cats with either confirmed or suspected salmonellosis (*2*). From September to October 2003, the New York State Department of Health and three local health departments identified seven human infections with *S. enterica* serovar Typhimurium, which exhibited an uncommon pulsed-field gel electrophoresis (PFGE) pattern. These cases had an apparent link to a veterinary clinic. This report describes the outbreak investigation and underscores the importance of integrating veterinary medicine into public health surveillance.

## The Study

In September 2003, five culture-positive human cases of *S*. Typhimurium infection were identified in three adjacent counties in New York State. All five isolates were indistinguishable by PFGE and were resistant to ampicillin, chloramphenicol, sulfisoxazole, streptomycin, and tetracycline. Onset dates were July 22 to August 22, 2003 (Figure). A local veterinary hospital, clinic X, was the only exposure common to all patients (Table). Patients 1 and 2 were veterinary technicians at clinic X, and patients 3–5 were pet owners whose pets had visited clinic X from July 15 to July 22, 2003. Laboratory surveillance identified two additional cases (cases 6 and 7) with matching PFGE patterns (Table, Figure). Symptoms of the infection included diarrhea, cramps, fever, and nausea. The median duration of illness was 8 days. A full investigation was conducted, including a site visit, case-finding, and diagnostic testing of clients and staff of clinic X.

**Figure:**
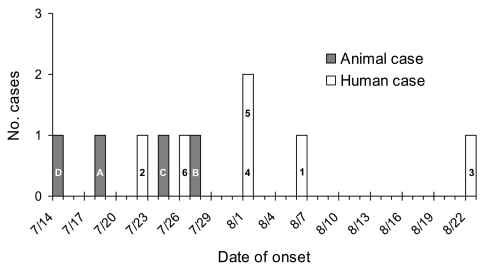
Epidemic curve of onset dates for human salmonellosis cases (white bars) and animal cases (gray bars). Numbers 1–6 refer to human cases, letters A–D refer to animal cases (see text).

**Table:** Human and animal *Salmonella enterica* serovar Typhimurium cases associated with a veterinary clinic, New York, 2003

Case	Age, sex/species	Possible exposures	Clinic date(s)^a^	Estimated onset date	Status^b^
Patient 1	31, F	Clinic technician		8/6	Confirmed
Patient 2	27, F	Clinic technician		7/22	Confirmed
Patient 3	64, F	Owner of pet A		8/22	Confirmed
Patient 4	2, F	Owner of pet B		8/1	Confirmed
Patient 5	93, F	Owner of pet C		8/1	Confirmed
Patient 6	2, M	Neighbor owns pet D		7/26	Confirmed
Patient 7	44, F	Unknown		9/14	Confirmed
Pet A	Cat	Dental work by patient 1	7/15–7/16, 7/23–7/25, 8/1	7/18	Suspected
Pet B	Cat	Dental work by patient 1	7/22–7/23, 7/27, 8/4	7/27	Confirmed
Pet C	Dog	Dental work by patient 1	7/22–7/23	7/24	Suspected
Pet D	Dog	In clinic for vomiting/diarrhea	7/14–7/15, 7/16–7/21	7/14	Suspected

^a^Dates when each animal was at clinic X. ^b^Confirmed, pulsed-field gel electrophoresis match on stool culture; suspected, clinical signs of enteritis or gastroenteritis, stool culture negative.

Interviews with clinic X staff and veterinary chart reviews determined that two cats and one dog owned by patients 3, 4, and 5 (pets A, B, and C, respectively) were admitted on two different dates in July for dental procedures (Table). All three procedures were performed by one veterinarian and one technician (patient 1). All three animals were held overnight, with evening treatments performed by patient 2, who works 1 evening per week. All procedures were performed in a designated room that was also used for other nonsterile procedures. The cats were held overnight in procedure room cages; the dog was held overnight either in the procedure room or in a dog run in a separate room.

All three animal patients were treated after the procedure with a prophylactic course of clindamycin. Pet B had a history of diabetes with chronic intermittent diarrhea attributed to diabetes-related dietary changes. The other pets had no prior illness. All three owners reported transient diarrhea in the pets after the dental procedure. Pet B developed severe mucoid diarrhea ≈5 days postsurgery and was treated with additional antibiotics (amoxicillin and enrofloxacin).

Patient 6 owns a dog but does not use clinic X. Patient 6 had occasional contact with his neighbor's dog (pet D), which had been to clinic X for several overnight visits because of severe vomiting and diarrhea attributed to eating mulch. Pet D was last discharged from clinic X on July 21, 5 days before patient 6's onset date. Whether patient 6 had contact with pet D between July 21 and July 26 is unclear.

The seventh patient was an emergency room nurse at a hospital in the outbreak area. She has a dog but does not use clinic X; the dog had no recent illness and had not recently been to a veterinarian. No other exposures to clinic X or other patients and pets could be identified. Stool culture of the patient's dog was negative.

After identification of the outbreak in September 2003, stool cultures were collected from the pets of all patients, including healthy contact pets from the same households. Only pet B, the diabetic cat, had a positive stool specimen collected at the end of September with a PFGE match to the human isolates.

Clinic X is a large, multidoctor practice that primarily treats dogs and cats, although one clinician (not linked to this outbreak) sees exotic animals, including reptiles. In addition to exam rooms, procedure room, and sterile surgery suite, the practice has separate rooms for isolation, animal wards, dog runs, surgery preparation, laboratory, reception, and patient files. No animals had been placed in isolation during the outbreak. A break room and meeting room are on a separate floor of the practice.

Thirty-seven of 38 uninfected staff members completed questionnaires regarding exposure and illness history. Seven reported diarrhea, and two reported nausea only between June 1 and August 31, 2003. None of the staff members submitted stool cultures. Stool culture from the asymptomatic veterinarian for the case-pets was negative, but patient 1 had continued signs of illness and was still culture-positive in mid-September. She voluntarily excluded herself from direct patient care until illness resolved.

A review of infection control practices in mid-September 2003 did not identify significant lapses in handwashing; cleaning; or disinfecting instruments, floors, or surfaces. No food was visible in the work areas during a walk-through, but the owner reported that he frequently reminds staff to avoid eating in work areas. Twenty-three environmental swabs were taken from the procedure room, anesthesia machines, animal wards (including isolation), and the laboratory (including a microscope used for fecal parasitology exams). Samples were collected by using sterile gauze sponges dampened with sterile double-strength skim milk. All were negative for *Salmonella*.

Clinic X staff telephoned dental clients treated since June 1, 2003 with a questionnaire developed by health department staff. The script asked about illness in pets or people in the household. No additional human or animal illnesses were identified.

## Conclusions

A likely source of *Salmonella* for this outbreak was not identified. The animal with the earliest illness onset (pet D) could have contaminated the clinic. However, pet D was not confirmed with *Salmonella* infection, was never in the dental room, and had no exposure to other case-pets. Another, unidentified animal patient may have been the source of contamination, or a person on the clinic staff may have been the source. If the clinic environment was the source of infection, cleaning apparently eliminated contamination by the time environmental specimens were collected in late September. Polymerase chain reaction (PCR) testing may have yielded different results; however, a positive result on a PCR test might represent nonviable bacterial DNA (*3**,**4*).

No epidemiologic link could be found to patient 7. However, out of 457 *Salmonella* isolates tested at Wadsworth Center Laboratories from January 2003 through July 2004, only the seven human patients and one cat reported here had this PFGE pattern. Patient 7 may have been exposed to undiagnosed cases through her work at the emergency room or through some other unidentified common exposure.

The outbreak described here was identified because the standard questionnaire used to interview patients included animal exposure questions, which shows the importance of animal exposure history in detecting potentially zoonotic diseases. This outbreak also shows the importance of zoonotic disease education for pet owners and increased awareness of zoonotic diseases by veterinarians. As is often the case with gastroenteritis in pets, the potential for transmission to humans was not considered. Since pet owners are frequently unwilling or unable to pay for diagnostic testing, veterinarians often do not consider stool culture for animals with diarrhea, which might have prevented some human cases in this outbreak. However, even without a definitive diagnosis, veterinarians can emphasize infection control practices with staff and educate owners that, although pets are rarely confirmed as the source of human salmonellosis, zoonotic transmission of gastrointestinal illnesses from sick pets may occur. Veterinarians should emphasize handwashing and infection control in the home. This practice is particularly important for households with immunocompromised persons or young children, who could become a source of secondary infection to other children, especially in daycare settings.

Finally, the importance of a good working relationship between public health and the veterinary community is underscored by the strong, cooperative relationship between public health authorities and clinic X. The clinic owner and staff were enlisted early as public health partners who took an active role in preventing further cases. Increasing concern about emerging zoonotic diseases and zoonotic agents as bioweapons has raised awareness of the risk of zoonotic disease exposure for persons employed in animal health. Agriculture, veterinary, and public health agencies in many states are promoting zoonotic disease awareness among veterinary professionals. As we continue to integrate the veterinary community into public health, information from these types of outbreaks should be used to develop protocols for zoonotic disease response and education in the veterinary and pet-owning communities.
